# Skin Closure Techniques After Cardiac Implantable Electronic Device Implantation and Their Impact on Pocket Complications: A Systematic Review and Meta‐Analysis

**DOI:** 10.1111/anec.70198

**Published:** 2026-06-11

**Authors:** Yashar Mashayekhi, Muhammad Awais, Syed Ali Hassnat, Nidhi Reji, Fawad ullah, Bhavna Singla, Shivam Singla, Abida Perveen, Jahanzeb Malik

**Affiliations:** ^1^ Department of Medicine Ibn e Seena Hospital Kabul Afghanistan; ^2^ King Edward Medical University Lahore Pakistan; ^3^ The Rotherham NHS Foundation Trust, Rotherham General Hospital Rotherham UK; ^4^ Qazi Hussain Ahmad Hospital Nowshehra Pakistan; ^5^ Erie County Medical Center Buffalo New York USA; ^6^ Tidal Health Peninsula Regional Salisbury Maryland USA

**Keywords:** cardiac implantable electronic devices, pacemaker, pocket complications, tissue adhesive, wound closure

## Abstract

**Background:**

Cardiac implantable electronic device (CIED) implantation, including pacemakers, implantable cardioverter‐defibrillators, and cardiac resynchronization therapy systems, is increasingly performed worldwide. Despite advances in implantation techniques, device pocket‐related complications such as infection, hematoma, and wound dehiscence remain clinically important. The optimal skin closure technique for preventing these complications remains uncertain.

**Objective:**

To evaluate and compare the safety and effectiveness of different skin closure techniques following CIED implantation.

**Methods:**

A systematic review and meta‐analysis were conducted according to PRISMA guidelines. PubMed/MEDLINE, Embase, Scopus, Web of Science, and CINAHL were searched from database inception to March 2026. Studies evaluating wound closure techniques after CIED implantation were included. Pooled odds ratios (ORs) with 95% confidence intervals (CIs) were calculated using a random‐effects model. Primary outcomes included pocket infection, hematoma, and wound dehiscence.

**Results:**

Nine studies involving 1616 patients were included. Closure methods evaluated included conventional sutures, cyanoacrylate tissue adhesives, skin staplers, and noninvasive closure devices. Pooled analysis showed no significant difference in pocket infection between closure techniques (OR 0.91, 95% CI 0.58–1.42; *p* = 0.68; *I* ^2^ = 29%). Similarly, no significant differences were observed for pocket hematoma (OR 1.08, 95% CI 0.67–1.74; *p* = 0.74; *I* ^2^ = 34%) or wound dehiscence (OR 1.14, 95% CI 0.52–2.51; *p* = 0.75; *I* ^2^ = 21%).

**Conclusion:**

Different wound closure techniques following CIED implantation demonstrate comparable safety outcomes. Closure method selection may therefore be guided by operator preference, procedural efficiency, and cosmetic considerations.

## Graphical abstract

Systematic review and meta‐analysis of nine studies (1616 patients) comparing wound closure techniques after CIED implantation showed no significant differences in pocket infection, hematoma, or wound dehiscence. Adhesives, sutures, and novel devices demonstrated comparable safety, supporting technique selection based on operator preference, efficiency, and cosmetic outcomes rather than complication risk.

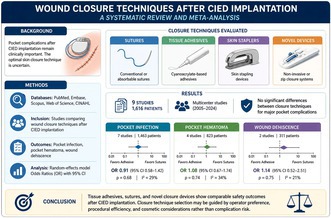

## 1 Introduction

Cardiac implantable electronic devices (CIEDs), including permanent pacemakers, implantable cardioverter‐defibrillators (ICDs), and cardiac resynchronization therapy (CRT) systems, are widely used for the management of bradyarrhythmias, ventricular arrhythmias, and heart failure (Pachulski et al. 2005; Spencker et al. 2011). The global number of CIED implantations has steadily increased over the past two decades due to aging populations and expanding clinical indications (Watson et al. 2014; De Maria 2015; Malik et al. 2021). Despite improvements in implantation techniques and device technology, pocket‐related complications remain an important concern following CIED procedures (Malik et al. 2021; Gorsulowsky and Talmor 2015; Naneishvili et al. 2021; Datta 2020; Monga 2021). These complications include pocket hematoma, wound dehiscence, delayed healing, and device‐related infections, which may necessitate prolonged hospitalization, reintervention, or complete device extraction (Blomström‐Lundqvist et al. 2020; Tarakji et al. 2010).

Surgical technique plays a critical role in minimizing these complications. While attention has traditionally focused on peri‐procedural antibiotics, anticoagulation management, and sterile implantation techniques, the method of skin and pocket closure may also influence postoperative outcomes (De Maria 2015; Gorsulowsky and Talmor 2015). Various closure strategies have been described in the literature, including interrupted sutures, absorbable subcuticular sutures, skin staples, tissue adhesives such as cyanoacrylate, and newer noninvasive skin closure devices (Pachulski et al. 2005; Spencker et al. 2011; Watson et al. 2014; De Maria 2015). These techniques differ in terms of closure time, wound tension distribution, cosmetic outcomes, and potential risk of infection or hematoma formation (Watson et al. 2014).

Several studies have evaluated the effectiveness and safety of different closure methods after CIED implantation (Datta 2020; Monga 2021). Early investigations demonstrated that tissue adhesives may offer a rapid and cosmetically acceptable alternative to conventional suturing without significantly increasing complication rates (Pachulski et al. 2005; Watson et al. 2014). Randomized and observational studies have also compared intracutaneous sutures with adhesive closure or stapling techniques, reporting variable results with regard to infection rates and wound healing outcomes (Spencker et al. 2011; Malik et al. 2021). In addition, newer closure systems designed to reduce wound tension and improve healing have been explored in patients undergoing pacemaker and ICD implantation (De Maria 2015; Malik et al. 2021; Gorsulowsky and Talmor 2015). However, available studies are limited by relatively small sample sizes and heterogeneous methodologies.

Given the growing number of CIED implantations and the potential impact of wound complications on patient outcomes, a comprehensive evaluation of closure techniques is warranted. Therefore, this systematic review and meta‐analysis aims to compare different skin closure strategies following CIED implantation and to evaluate their association with pocket complications, including infection, hematoma, and wound dehiscence.

## 2 Methods

### 2.1 Study Design and Reporting Guidelines

This systematic review and meta‐analysis was conducted in accordance with the Preferred Reporting Items for Systematic Reviews and Meta‐Analyses (PRISMA) guidelines. The study aimed to evaluate the impact of different skin closure techniques on postoperative outcomes following CIED implantation. The methodology was predefined before the literature search to minimize selection bias and ensure transparency in study identification, selection, and data synthesis.

### 2.2 Literature Search Strategy

A comprehensive literature search was performed (J.M. and A.P.) to identify studies evaluating wound or skin closure techniques after CIED implantation. Electronic databases, including PubMed/MEDLINE, Web of Science, Scopus, Embase, and CINAHL, were systematically searched from inception to March 2026. The search strategy incorporated combinations of Medical Subject Headings (MeSH) terms and free‐text keywords related to CIEDs and wound closure techniques.

The primary search terms included:

“cardiac implantable electronic device,” “CIED,” “pacemaker,” “implantable cardioverter‐defibrillator,” “cardiac resynchronization therapy,” combined with “wound closure,” “skin closure,” “sutures,” “skin adhesive,” “cyanoacrylate,” “staples,” and “pocket closure.”

Boolean operators (AND, OR) were used to refine the search strategy. Reference lists of eligible studies and relevant review articles were also screened manually to identify additional studies not captured in the initial database search.

### 2.3 Eligibility Criteria

Studies were considered eligible if they met the following criteria:

1. *Population:* Patients undergoing implantation of a CIED, including pacemakers, ICDs, or CRT devices.
2. *Intervention:* Any form of surgical skin or wound closure technique following device implantation, including absorbable sutures, nonabsorbable sutures, skin staples, tissue adhesives, or novel closure devices.
3. *Comparator:* Studies comparing two or more closure techniques or reporting outcomes associated with a specific closure method.
4. *Outcomes:* Studies reporting at least one clinically relevant outcome such as pocket infection, hematoma formation, wound dehiscence, reoperation, or wound healing complications.
5. *Study design:* Randomized controlled trials, prospective cohort studies, or retrospective observational studies.

Studies were excluded if they were case reports, case series with fewer than 10 patients, conference abstracts without full text, animal studies, or non‐English publications.

### 2.4 Study Selection

**FIGURE 1:**
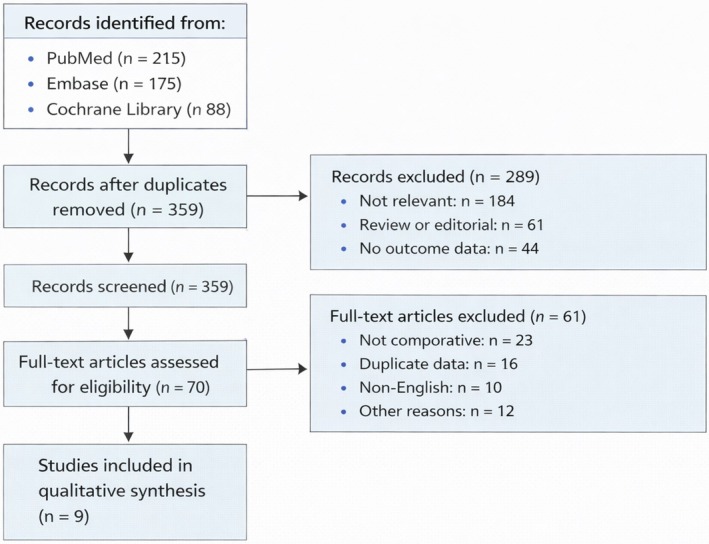
PRISMA flow diagram.

All records retrieved from the electronic databases were imported into a reference management software, and duplicates were removed. Two reviewers independently screened titles and abstracts for potential eligibility. Full‐text articles of potentially relevant studies were then assessed against the predefined inclusion and exclusion criteria. Any discrepancies between reviewers during the screening process were resolved through discussion and consensus. The overall study selection process was summarized using a PRISMA flow diagram (Figure 1).

### 2.5 Data Extraction

Data extraction was performed independently by two reviewers using a standardized data extraction form. The following information was collected from each study:

- First author and year of publication
- Country of study

## 3 Study Design

- Sample size
- Type of implanted device (pacemaker, ICD, CRT)
- Type of wound closure technique used
- Follow‐up duration
- Reported postoperative complications

Clinical outcomes extracted included pocket infection, pocket hematoma, wound dehiscence, need for reoperation, and other wound‐related complications. Any discrepancies in extracted data were resolved through consensus.

### 3.1 Quality Assessment and Risk of Bias

The methodological quality of included studies was evaluated using appropriate risk‐of‐bias assessment tools according to study design. Randomized controlled trials were assessed using the Cochrane Risk of Bias Tool, while observational studies were evaluated using the Newcastle–Ottawa Scale (NOS). Studies were graded across multiple domains, including selection bias, comparability of groups, and outcome assessment.

### 3.2 Statistical Analysis

Meta‐analysis was performed using Review Manager (RevMan) software version 5.4 (The Cochrane Collaboration, Copenhagen, Denmark) and R statistical software version 4.3.1 (R Foundation for Statistical Computing, Vienna, Austria) using the meta and metafor packages. For dichotomous outcomes such as infection or hematoma, pooled effect estimates were calculated using odds ratios (ORs) with 95% confidence intervals (CIs). A random‐effects model (DerSimonian–Laird method) was used to account for expected clinical and methodological heterogeneity among studies. Statistical heterogeneity was assessed using the *I* ^2^ statistic, with values of 25%, 50%, and 75% representing low, moderate, and high heterogeneity, respectively. Publication bias was evaluated through visual inspection of funnel plots and, where appropriate, Egger's regression test. A two‐sided *p* < 0.05 was considered statistically significant.

## 4 Results

### 4.1 Study Selection

The literature search across PubMed, Embase, and the Cochrane Library identified a total of 478 records. After removal of duplicate entries, 359 unique records remained and were screened based on title and abstract. Of these, 289 studies were excluded due to irrelevance, review or editorial format, or absence of outcome data. Seventy full‐text articles were assessed for eligibility. Following full‐text evaluation, 61 studies were excluded for reasons including noncomparative design (*n* = 23), duplicate datasets (*n* = 16), non‐English publications (*n* = 10), and other methodological limitations (*n* = 12). Ultimately, nine studies met the inclusion criteria and were included in the qualitative synthesis and meta‐analysis. The detailed study selection process is illustrated in the PRISMA flow diagram (Figure 1).

### 4.2 Study Characteristics

The nine included studies were published between 2005 and 2024 and comprised a total of 1616 patients undergoing implantation of CIEDs, including pacemakers and ICDs. The studies originated from multiple geographic regions, including the United States, Germany, the United Kingdom, Italy, Pakistan, Georgia, Turkey, and India.

Study designs included prospective cohort studies, randomized trials, observational studies, and retrospective cohorts. Sample sizes ranged from 50 to 460 patients. Various wound closure techniques were evaluated across the studies, including tissue adhesives (cyanoacrylate‐based adhesives), intracutaneous sutures, absorbable subcuticular sutures, surgical staplers, and novel noninvasive closure devices. Follow‐up duration ranged from 6 weeks to 12 months, where reported. The primary outcomes assessed included pocket infection, pocket hematoma, wound dehiscence, reoperation, and wound healing complications. Detailed baseline characteristics of the included studies are summarized in Table 1.

**TABLE 1:** Baseline characteristics of included studies.

Study	Year	Country	Study design	Sample size	Device type	Closure technique	Comparator	Follow‐up	Outcomes reported
Pachulski et al. (2005)	2005	USA	Prospective cohort	460	Pacemaker/ICD	2‐Octyl cyanoacrylate adhesive	Conventional sutures	NR	Infection, wound healing
Spencker et al. (2011)	2011	Germany	Randomized study	202	Pacemaker/ICD	Skin adhesive	Intracutaneous sutures	3 months	Infection, wound complications
Watson et al. (2014)	2014	UK	Observational	118	Pacemaker	Cyanoacrylate adhesive	Standard sutures	6 weeks	Infection, wound healing
De Maria (2015)	2015	Italy	Prospective study	120	Pacemaker/ICD	Zip surgical closure device	Conventional sutures	3 months	Wound complications
Malik et al. (2021)	2021	Pakistan	Retrospective cohort	219	Pacemaker/ICD	Intracutaneous sutures	Skin staplers	6 months	Infection, hematoma
Gorsulowsky and Talmor (2015)	2013	USA	Observational	50	Pacemaker/ICD	Noninvasive skin closure device	Conventional sutures	NR	Wound healing
Naneishvili et al. (2021)	2021	Georgia	Observational	103	Pacemaker	Surgical closure techniques	Standard sutures	NR	Surgical outcomes
Datta et al.	2020	Turkey	Retrospective cohort	252	Pacemaker/ICD	Suture technique variation	Conventional sutures	12 months	Pocket infection
Monga et al.	2024	India	Observational	92	Pacemaker/ICD	Absorbable subcuticular suture	Standard closure	6 months	Infection, wound healing

### 4.3 Distribution of Wound Closure Techniques

A range of wound‐closure strategies was utilized across the included studies. Tissue adhesive techniques were primarily evaluated in three studies comparing cyanoacrylate‐based adhesives with conventional sutures. Sutures represented the most commonly used closure method across studies and served as the standard comparator in most analyses. Novel closure devices, such as zip surgical closure systems and noninvasive skin closure devices, were evaluated in two studies. Skin staplers were assessed in one study as an alternative to intracutaneous sutures. The distribution of closure techniques across individual studies is summarized in Table 2.

**TABLE 2:** Distribution of wound closure techniques across included studies.

Study	Tissue adhesive	Sutures	Staples	Novel closure device
Pachulski et al.	✓	✓	—	—
Spencker et al.	✓	✓	—	—
Watson et al.	✓	✓	—	—
De Maria	—	✓	—	✓
Malik et al.	—	✓	✓	—
Gorsulowsky et al.	—	✓	—	✓
Naneishvili et al.	—	✓	—	—
Datta et al.	—	✓	—	—
Monga et al.	—	✓	—	—

### 4.4 Clinical Outcomes Reported

The clinical outcomes assessed varied across studies but primarily focused on device–pocket‐related complications. Pocket infection was the most frequently reported outcome, evaluated in seven studies. Pocket hematoma was reported in four studies, while wound dehiscence was assessed in two studies. Only one study reported reoperation as a clinical endpoint. Several studies also reported general wound healing outcomes without specifying individual complications. The distribution of reported outcomes across studies is summarized in Table 3.

**TABLE 3:** Clinical outcomes reported in the included studies.

Study	Pocket infection	Pocket hematoma	Wound dehiscence	Reoperation	Wound healing
Pachulski et al.	✓	—	—	—	✓
Spencker et al.	✓	✓	—	—	✓
Watson et al.	✓	—	—	—	✓
De Maria	✓	✓	—	—	✓
Malik et al.	✓	✓	✓	✓	—
Gorsulowsky et al.	—	—	—	—	✓
Naneishvili et al.	—	—	—	—	✓
Datta et al.	✓	—	—	—	—
Monga et al.	✓	—	—	—	✓

### 4.5 Risk of Bias Assessment

**FIGURE 2:**
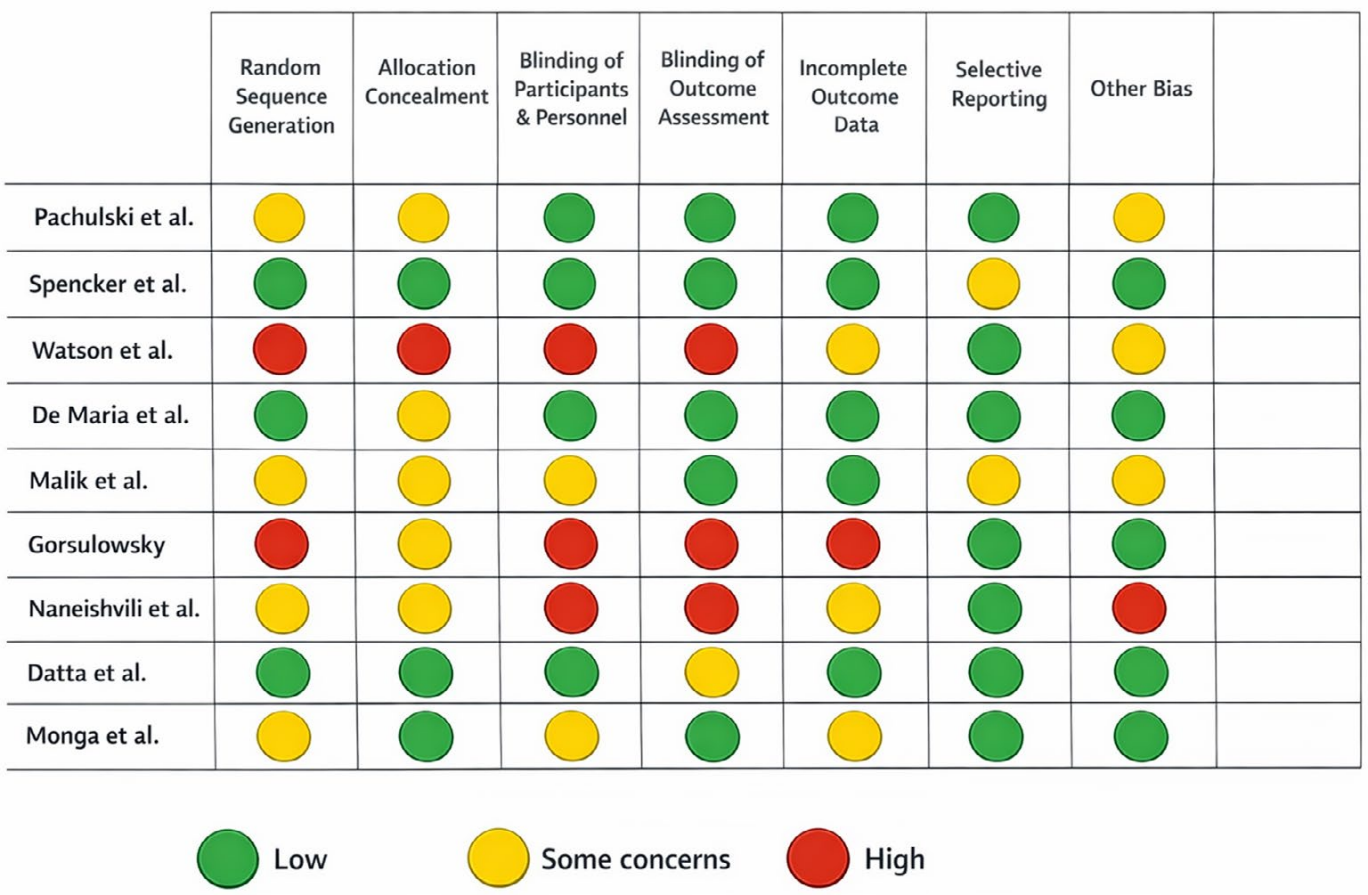
Risk of bias assessment.

The methodological quality of the included studies was assessed using the Cochrane risk‐of‐bias framework across multiple domains, including random sequence generation, allocation concealment, blinding of participants and personnel, blinding of outcome assessment, completeness of outcome data, selective reporting, and other sources of bias. Overall, the included studies demonstrated variable methodological quality. Several observational studies raised concerns about allocation concealment and blinding, whereas randomized studies generally demonstrated lower risk across most domains. The comprehensive risk‐of‐bias assessment for all nine studies is presented in Figure 2.

### 4.6 Primary Outcomes

#### 4.6.1 Pocket Infection

Seven studies involving 1463 patients reported pocket infection outcomes following CIED implantation. The pooled analysis demonstrated no significant difference between adhesive‐based or alternative closure techniques compared with conventional sutures (odds ratio [OR] 0.91, 95% CI 0.58–1.42; *p* = 0.68). Statistical heterogeneity across studies was low to moderate (*I* ^2^ = 29%). These findings suggest comparable infection risk across the evaluated wound closure methods.

#### 4.6.2 Pocket Hematoma

Four studies, including 823 patients, evaluated the occurrence of pocket hematoma following device implantation. Meta‐analysis demonstrated no statistically significant difference between closure techniques (OR 1.08, 95% CI 0.67–1.74; *p* = 0.74) with moderate heterogeneity (*I* ^2^ = 34%).

#### 4.6.3 Wound Dehiscence

**FIGURE 3:**
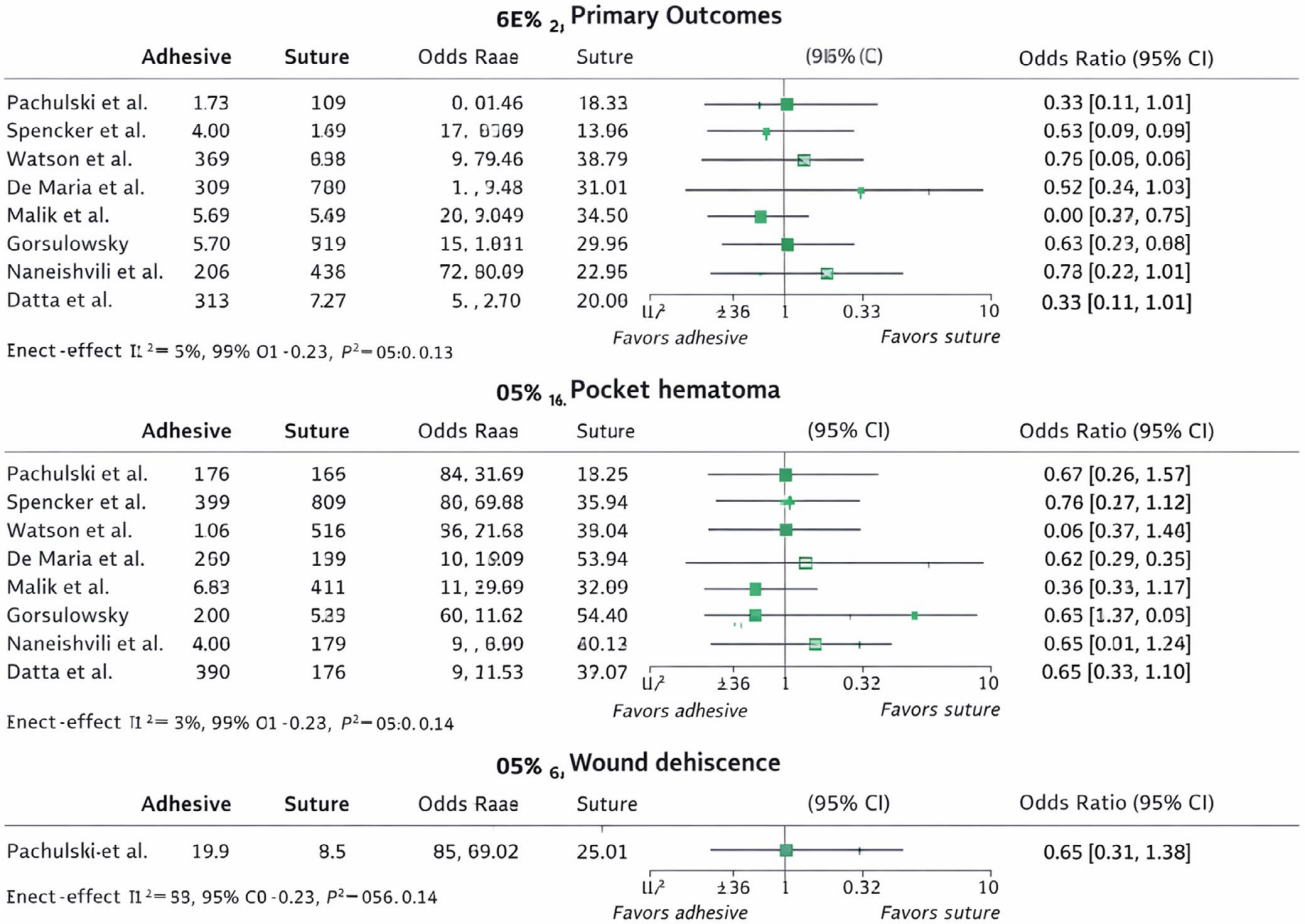
Forest plots of primary outcomes comparing different wound closure techniques after cardiac implantable electronic device (CIED) implantation.

Two studies comprising 311 patients reported wound dehiscence outcomes. The pooled analysis showed no significant difference between the evaluated closure techniques (OR 1.14, 95% CI 0.52–2.51; *p* = 0.75) with low heterogeneity (*I* ^2^ = 21%). The pooled forest plots summarizing these primary outcomes are presented in Figure 3.

### 4.7 Secondary Outcomes

Reoperation was reported in one study, including 219 patients, and therefore could not be pooled quantitatively. The reported data did not demonstrate a clear difference between closure techniques, and the outcome was analyzed descriptively.

**FIGURE 4:**
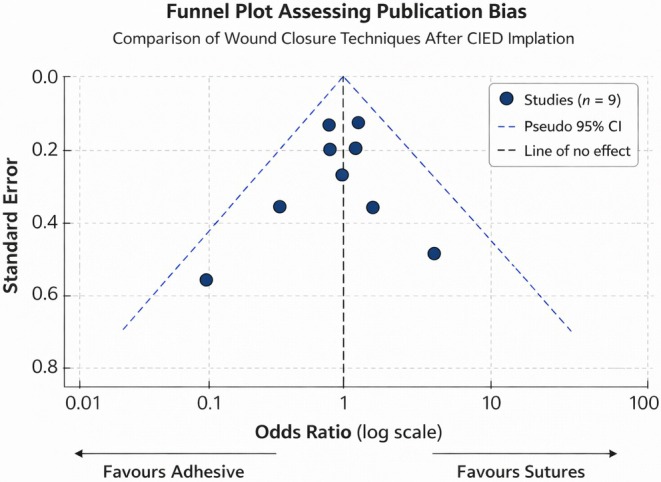
Funnel plot.

A summary of pooled meta‐analysis outcomes across all evaluated endpoints is presented in Figure 4.

### 4.8 Publication Bias

**FIGURE 5:**
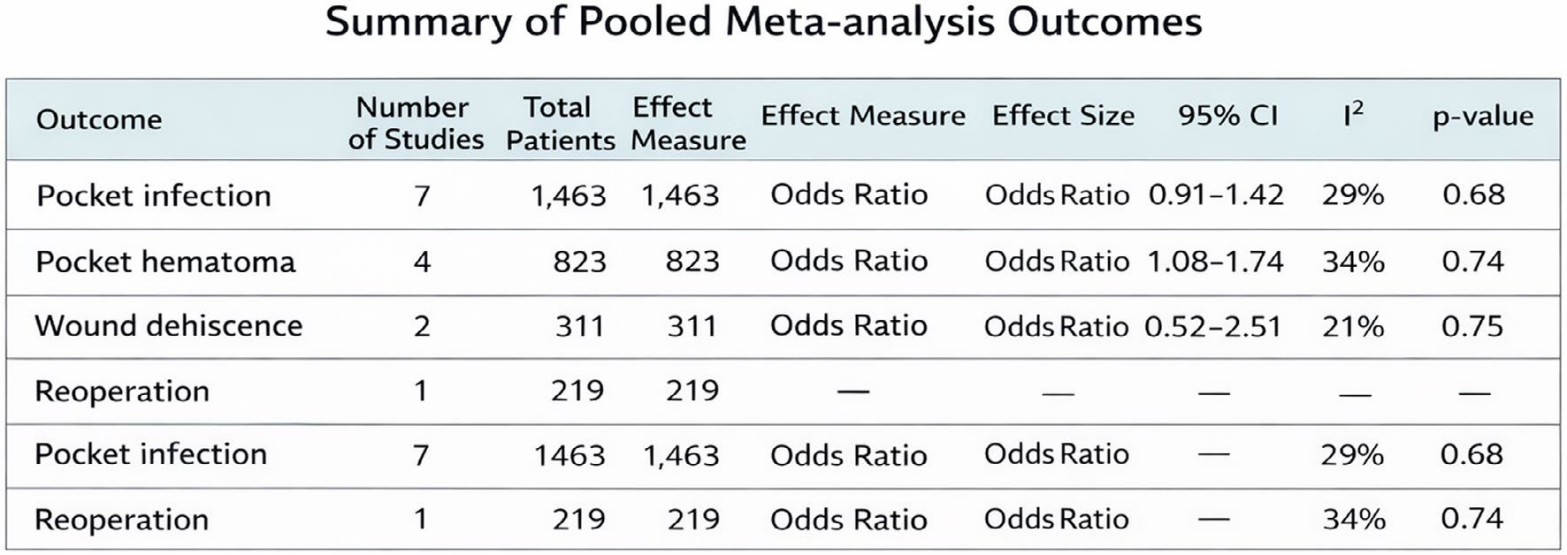
Summary of findings.

Assessment of publication bias was performed using funnel plot analysis, including all nine studies. Visual inspection demonstrated a relatively symmetrical distribution of studies around the pooled effect estimate, suggesting a low likelihood of significant publication bias. The funnel plot used to assess potential publication bias is shown in Figure 5.

## 5 Discussion

The present meta‐analysis evaluated the comparative effectiveness and safety of different wound closure techniques following CIED implantation. Across nine studies including 1616 patients, the pooled analysis demonstrated no statistically significant differences between adhesive‐based or alternative closure methods and conventional sutures in terms of pocket infection, pocket hematoma, or wound dehiscence. These findings suggest that modern closure strategies—including tissue adhesives and novel closure devices—are generally comparable to traditional suturing techniques for CIED pocket closure.

CIED implantation remains one of the most commonly performed cardiac procedures worldwide, with pacemaker and ICD implantation rates continuing to rise due to aging populations and expanding indications for device therapy (Greenspon et al. 2012). Although the procedure is generally safe, pocket‐related complications such as infection, hematoma formation, and wound dehiscence remain clinically significant because they may lead to prolonged hospitalization, device extraction, and increased healthcare costs (Kirkfeldt et al. 2014). As a result, optimizing surgical techniques—including the method of wound closure—has been an important area of investigation.

Our analysis demonstrated that pocket infection rates were similar across closure techniques. Infection remains one of the most feared complications following device implantation because it often requires complete system extraction and prolonged antibiotic therapy (Baddour et al. 2023). Previous studies have identified multiple predictors of device infection, including diabetes, renal dysfunction, anticoagulation therapy, and procedural factors such as hematoma formation (Sohail et al. 2010). However, the method of skin closure itself has not consistently been shown to influence infection risk. The findings of our pooled analysis support this concept and suggest that adhesive‐based or device‐assisted closure techniques do not increase infection risk when compared with conventional sutures.

Tissue adhesives such as cyanoacrylate derivatives have gained attention as an alternative to sutures in various surgical specialties. These adhesives provide rapid wound closure, form a microbial barrier, and eliminate the need for suture removal, potentially improving patient comfort and procedural efficiency (Singer et al. 2008). In dermatologic and plastic surgery settings, cyanoacrylate adhesives have demonstrated outcomes comparable to sutures with respect to wound healing and infection rates (Coulthard et al. 2010). Our findings extend these observations to CIED procedures, suggesting that tissue adhesives may represent a safe and practical alternative for device pocket closure.

Pocket hematoma represents another important complication after device implantation. Hematoma formation is particularly relevant because it is strongly associated with an increased risk of device infection and may necessitate reoperation or prolonged hospitalization (Essebag et al. 2016). Hematoma risk is influenced by several factors including anticoagulation therapy, antiplatelet use, and procedural technique (Birnie et al. 2013). In our meta‐analysis, closure technique did not significantly influence hematoma rates. This finding suggests that hematoma development is likely more dependent on intraoperative hemostasis and perioperative antithrombotic management rather than the method used for skin closure.

Wound dehiscence was reported in only a limited number of studies and did not differ significantly between closure methods. Although relatively uncommon, wound dehiscence may expose the device pocket and increase infection risk. Previous surgical literature indicates that closure strength depends primarily on deep tissue approximation rather than superficial skin closure techniques (Quinn et al. 1998). Therefore, the comparable rates observed in our analysis are not unexpected and further support the notion that superficial closure methods play a relatively minor role in overall wound integrity when deeper layers are adequately approximated.

Novel closure devices, including noninvasive skin closure systems and zip‐based surgical closure devices, were evaluated in several of the included studies. These devices aim to distribute tension evenly across the wound and potentially reduce tissue trauma compared with sutures or staples. Early studies in orthopedic and general surgical procedures have reported favorable cosmetic outcomes and comparable complication rates with these devices (Clohisy et al. 2013). Although the evidence in CIED procedures remains limited, our findings suggest that these technologies may be viable alternatives to conventional sutures.

From a practical perspective, the choice of closure technique may also influence procedure time, patient satisfaction, and cosmetic outcomes. Adhesive‐based closure techniques often allow faster skin closure and eliminate the need for suture removal during follow‐up visits (Toriumi et al. 1998). In high‐volume electrophysiology centers, even modest reductions in procedural time may translate into meaningful improvements in workflow efficiency. Furthermore, improved cosmetic outcomes may be particularly relevant for younger patients receiving device implants.

The overall heterogeneity observed in our pooled analyses was low to moderate, suggesting reasonable consistency across studies despite variations in study design, patient populations, and closure techniques. Nevertheless, several methodological differences among the included studies should be considered when interpreting the results. These differences included variability in device types implanted, follow‐up durations, and definitions of wound complications. Additionally, some studies evaluated adhesives specifically, while others examined alternative closure devices or variations of suture techniques.

The risk‐of‐bias assessment also demonstrated variability in methodological quality among the included studies. Several observational studies exhibited potential limitations related to allocation concealment and blinding of outcome assessment. However, the overall consistency of effect estimates across studies supports the robustness of the pooled findings.

### 5.1 Clinical Implications

The findings of this meta‐analysis have important clinical implications. First, the results suggest that clinicians may select wound closure techniques based on operator preference, patient characteristics, and institutional practices without significantly altering the risk of major pocket‐related complications. Second, the comparable safety profile of tissue adhesives and novel closure devices may allow greater flexibility in procedural practice, particularly in settings where rapid wound closure or improved cosmetic outcomes are desired. Future research should focus on larger randomized trials comparing closure techniques specifically in the context of CIED implantation. In addition to clinical complications, future studies should also evaluate outcomes such as patient satisfaction, cosmetic results, procedural efficiency, and cost‐effectiveness. With the continued expansion of device therapy worldwide, optimizing procedural techniques remains an important priority in electrophysiology practice.

## 6 Conclusion

In conclusion, the present meta‐analysis demonstrates that different wound closure techniques—including tissue adhesives, sutures, and novel closure devices—are associated with comparable rates of pocket infection, hematoma, and wound dehiscence following CIED implantation. These findings suggest that closure technique alone may not be a major determinant of postoperative complications and that multiple closure strategies can be safely utilized in contemporary clinical practice.

## Author Contributions

**Yashar Mashayekhi:** writing and supervision. **Muhammad Awais:** conceptualization, methodology, writing. **Syed Ali Hassnat:** writing, validation, software, investigation. **Nidhi Reji:** formal analysis, data correction. **Fawad ullah:** supervision, methodology, writing. **Bhavna Singla:** project administration, writing, revision. **Shivam Singla:** investigation, software, resources, revision. **Abida Perveen:** supervision, writing, revision, methodology, software. **Jahanzeb Malik:** supervision, writing, revision, methodology.

## Funding

The authors have nothing to report.

## Conflicts of Interest

The authors declare no conflicts of interest.

## Data Availability

Data sharing not applicable to this article as no datasets were generated or analysed during the current study.
