# Correction to “Skin Closure Techniques After Cardiac Implantable Electronic Device Implantation and Their Impact on Pocket Complications: A Systematic Review and Meta‐Analysis”

**DOI:** 10.1111/anec.70246

**Published:** 2026-10-23

Mashayekhi, Y. , M. Awais , S. A. Hassnat , et al. 2026. “Skin Closure Techniques After Cardiac Implantable Electronic Device Implantation and Their Impact on Pocket Complications: A Systematic Review and Meta‐Analysis.” Annals of Noninvasive Electrocardiology 31, no. 4: e70198. 10.1111/anec.70198.PMC1325514342274220

The affiliation of the first author, Yashar Mashayekhi has been corrected. The correct affiliation is as follows:

Department of Orthopaedics, Leicester University Hospitals, Leicester, UK.

We apologize for this error.

